# Evaluation of L6 augmentation signal reception characteristics and positioning accuracy of compact and lightweight GNSS antennas

**DOI:** 10.1038/s41598-023-48954-0

**Published:** 2023-12-08

**Authors:** Taro Suzuki

**Affiliations:** Chiba Institute of Technology, Future Robotics Technology Center, 2-17-1, Tsudanuma, Narashino, Chiba, 2750016 Japan

**Keywords:** Aerospace engineering, Civil engineering

## Abstract

Applications requiring outdoor position estimation, such as unmanned construction and delivery automation, focus on receiving global navigation satellite system (GNSS) correction information from satellites for high-precision positioning. In particular, the delivery of correction information for the Galileo high-accuracy service (HAS) and quasi-zenith satellite system (QZSS) centimeter-level augmentation service (CLAS) is based on a new frequency band called L6. The L6 signal is a new type of GNSS signal, and a GNSS antenna corresponding to the frequency of the L6 signal (1275.46 MHz) is required to receive and decode the correction messages. The reception characteristics of the L6 signal are important for receiving correction information. However, the reception performance of antennas supporting the new L6 signal has not been evaluated. Therefore, in this study, we evaluate the reception characteristics of the L6 signal of a compact and lightweight L6-compatible antenna, and the multipath characteristics, which are the fundamental performance of the antenna that affects high-precision positioning. In a 24-hour static test, each antenna’s signal reception performance and multipath characteristics were evaluated, and significant differences were found in performance among the antennas capable of receiving the L6 signal. Furthermore, in a kinematic test, we evaluated high-accuracy positioning using QZSS CLAS with multiple antennas and showed that centimeter-level positioning using L6 augmentation signals is possible even with compact and lightweight GNSS antennas. These evaluations provide guidelines for antenna selection when high-precision positioning using L6 signals is employed in various applications.

## Introduction

Global navigation satellite systems (GNSSs) are widely used in various outdoor applications as position estimation methods. In particular, highly accurate real-time positional information in outdoor environments is required for the automation of construction and agricultural machinery, delivery by drones and ground robots, and automated vehicle operation. Achieving centimeter-level positioning with GNSS requires correction for various errors in GNSS observations, such as ionospheric and tropospheric delays, satellite orbit errors, and satellite clock errors. Typical high-precision positioning methods include real-time kinematic (RTK) GNSS, which uses the double difference between the GNSS reference station and the GNSS observation of a user, and precise point positioning (PPP), which provides centimeter-level positioning accuracy with a single receiver, using precise satellite orbit and clock products generated by a network of GNSS reference stations distributed around the world^1^. These methods are widely used in the above applications; however, the main difficulty with these methods is obtaining the correction information. In RTK-GNSSs, local radio communication with a reference station or networked RTK-GNSSs using the Internet is widely used. In PPP, real-time positioning can be achieved by receiving correction information over an internet connection.

However, a method that uses a local area network incurs a large system construction cost. Methods that use the Internet cannot be used in environments where Internet access is not available, e.g., in the mountains or at sea. Therefore, high-precision positioning methods for distributing satellite correction information have attracted considerable attention in recent years. Trimble RTX^2^, TerraStar^3^, and u-blox PointPerfect^4^ are well-known commercial services. These services receive correction information from geostationary satellites by using L-band frequencies to achieve high-precision positioning. These services are fee-based and require separate agreements. On the other hand, GNSS correction information distribution services that can be used free of charge include MADOCA-PPP^5^, which provides correction information via quasi-zenith satellite system (QZSS), and centimeter-level augmentation service (CLAS)^6^, which provides correction information for PPP-RTK launched in 2021 by the Japanese QZSS. In addition, European Galileo launched a high-precision positioning service (HAS)^7^ in 2022. Common to all these services is providing PPP or PPP-RTK correction information from a satellite and using the L6 band (1275.46 MHz). The QZSS MADOCA, CLAS, and Galileo HAS messages are transmitted over signals called L6E, L6D, and E6. A GNSS antenna corresponding to the L6 band frequency and a GNSS receiver capable of tracking the L6D/E and E6 signals are required to use these corrected information.

The following problems occur when high-precision positioning services are used with an L6 signal:

- The reception characteristics of the L6 signal are important for receiving correction information from satellites in real-world environments; however, the reception performance of the L6 signal, especially with compact and lightweight antennas supporting the new L6 signal has not been adequately evaluated.
- Compact and lightweight antennas are suitable for the high-precision positioning of construction machinery, agricultural machinery, robots, and vehicles. The correction signal transmitted at L6 is usually applied to the GNSS observations of the L1 or L2 signal. However, compact and lightweight antennas often have lower signal reception strength, multipath characteristics, and carrier phase center stability compared with surveying antennas. Moreover, the performance of small and lightweight antennas has not been evaluated, and whether they can be applied to high-precision positioning using L6 signals is not clear.

As the L6 signal is close in frequency to the conventionally used GNSS L2 signal^8^, the L6 signal can often be received at a high signal strength (at high elevation angles) on an antenna capable of receiving L2. However, some antennas cannot receive L6 signals at low elevation angles even though they are designed to support the L6 band. Therefore, an evaluation of L6-compatible antennas must be conducted.

This study evaluates the reception characteristics of the L6 signal of a compact and lightweight L6-compatible antenna that can be used in robots, and the multipath characteristics, which are the fundamental performance of the antenna that affects high-precision positioning. This provides guidelines for antenna selection when using the QZSS CLAS, MADOCA-PPP, or Galileo HAS for high-precision positioning in real-world applications.

## Related studies

Several studies have been conducted to evaluate the performance of GNSS antennas in terms of their signal reception capabilities, including their multipath immunity^9,10^. These studies have focused on the calibration of the antenna carrier phase center and its variation^11^ and on evaluating how the antenna phase center calibration parameters affect positioning accuracy^12^. Historically, antenna evaluation research has primarily focused on large survey-grade antennas.

Recently, several low-cost multi-frequency GNSS antennas have become available with the proliferation of low-cost dual-frequency GNSS receivers. Moreover, the performance of small, lightweight, and low-cost GNSS antennas has rarely been evaluated. Hamza et al. (2021)^13^ and Krietemeyer et al. (2020)^14^ evaluated the accuracy of GNSS observations and the positioning accuracy of low-cost dual-frequency GNSS receivers, survey GNSS antennas, and small patch antennas. Hamza et al.^15^, Poluzzi et al. (2020)^16^ compared the RTK-GNSS accuracies of three low-cost antennas. In recent years, low-cost antennas for smartphones have been evaluated, as smartphones can be used to acquire raw GNSS measurements^17,18^. However, these studies only evaluated antennas for the GNSS L1 signal and not for the L6 signal. Moreover, these low-cost antennas have only been evaluated as patch antennas, and few evaluations have been conducted on helical GNSS antennas, which have become popular in recent years. Li et al. (2022)^19^ compared the positioning accuracy of helical and surveying antennas in smartphones; however, the performance of helical antennas for L6 signals was not evaluated. Karasawa et al.^20^ evaluated the reception capability of the developed L6 antenna; however, no detailed comparison with existing antennas was made.

On the other hand, PPP and PPP-RTK have been evaluated using L6 signal augmentation data. Suzuki et al. (2014)^21^ evaluated PPP using QZSS MADOCA, and^22^ evaluated PPP-RTK using QZSS CLAS for their positioning accuracy in a real outdoor environment. Fernandez-Hernandez et al. (2023)^23^ and Naciri et al. (2023)^24^ evaluated positioning accuracy using Galileo’s HAS. However, all these evaluations of high-precision positioning using the L6 signal used survey antennas; whether compact and lightweight antennas can be used for high-precision positioning using the L6 signal is still not clear.

This study evaluates the reception performance of L6 signals and the multipath performance of several compact, lightweight antennas. We also compare the positioning performance of these antennas using L6 augmentation signal in real-world conditions using kinematic tests.

## Antenna evaluation method

**Figure 1:**
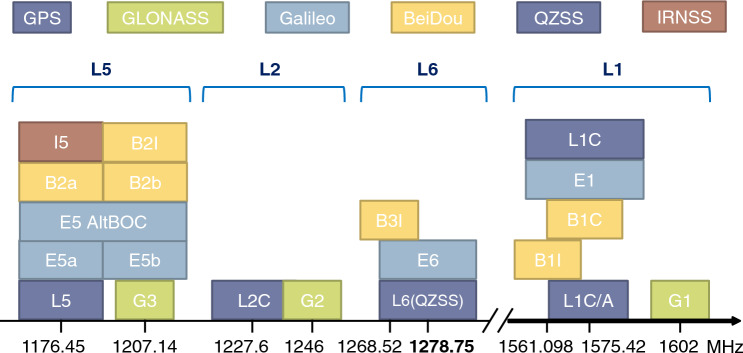
Relationship between GNSS transmission signal type and its frequency. The center frequency of the L6 signal is 1278.75 MHz, which is close to the G2 signal frequency of GLONASS, which has been widely used for a long time.

Figure 1 shows the relationship between the main GNSS frequencies and types of transmitted signals. The L6 frequency band is close to the L2 band, which has been widely used in GNSS receivers for a long time and is the most recently used transmission frequency compared to other signals. The L6 frequency band is approximately 30 MHz away from the G2 signal, which is the L2 signal of GLONASS. In many cases, antennas that can receive the L2 signal can also receive the L6 signal, which is close in frequency to the L2 signal, although the received signal strength is reduced. Therefore, even the antennas listed in the catalog as being capable of receiving L6 signals fail to receive them from satellites at low elevation angles, which is a practical problem. In this study, we evaluate L6-compatible GNSS antennas in terms of the following factors.

### Size and weight

For delivery robots and drones, the size and weight of the antenna are important selection criteria because of the weight that can be carried and space limitations. Here, antennas with a weight of 250 g or less and a diameter of 100 mm or less were selected as L6-compatible antennas for evaluation. Compact and lightweight GNSS antennas can be used for various applications. However, smaller antennas generally have lower gain and poorer multipath performance than survey-grade antennas^9^. Therefore, this study compares the performance of a small, lightweight, L6-compatible antenna with that of survey-grade antennas.

### Antenna type

Two types of GNSS antenna systems are commonly used to receive circularly polarized GNSS signals: patch antennas, which consist of plate-shaped conductors with additional feed points stacked at different frequencies, and helical antennas, which consist of spiral-shaped conductors. Patch antennas have been used for a long time in GNSS surveys. Helical antennas are significantly lighter than patch antennas. Therefore, helical GNSS antennas, such as drones, have been widely used in recent years for applications with strict onboard weight restrictions. In addition, helical antennas are smaller in radial size than patch antennas but generally taller. This study also compared the performances of patch and helical antennas.

### Phase center information

A phase-center offset (PCO) relative to the antenna reference point (ARP) is required to use the antenna for precise survey applications. For surveying applications, antenna calibration must further correct the phase-center variation (PCV) corresponding to the satellite elevation angle and frequency. However, PCV correction through calibration is rarely provided for small, lightweight, and inexpensive antennas, and some antennas do not provide PCO for ARP. The availability of an antenna’s phase-center information is very important when applying L6-based high-precision positioning to various applications.

### Elevation angle: L6 signal strength characteristics

As mentioned above, the L6 signal strength is crucial for high-precision positioning applications. A low signal strength causes unstable signal tracking and prevents the decoding of L6 signal messages. Therefore, this study evaluates each antenna’s L6 signal reception capability by evaluating the actual L6 signal strength versus the elevation angle in an open-sky environment.

### Multipath characteristics

In the case of Galileo HAS and QZSS CLAS and MADOCA, the correction signal transmitted at L6 signal is applied to the GNSS observations of the L1, L2 or L5 signals. When PPP and RTK are used for high-accuracy positioning, the multipath characteristics of the pseudorange and carrier phase of the antenna are very important. If the multipath characteristics of the antenna are poor, the positioning error will increase and the carrier phase ambiguity fixed rate in the PPP-RTK will decrease. Therefore, in this study, we evaluate the magnitude of the pseudorange multipath error in the L1 signal for each small antenna using linear multipath combinations to evaluate the multipath characteristics of the antennas.

### Positioning accuracy

For a comprehensive evaluation of L6-compatible antennas, we received QZSS L6D signals under real-world conditions and evaluated the positioning accuracy of PPP-RTK using CLAS. Using the high-precision positioning method with L6 signals, we evaluated whether, in a real environment, the survey and small or light antennas showed any difference in the positioning accuracy and fixed rate.

## Target antennas

**Figure 2:**
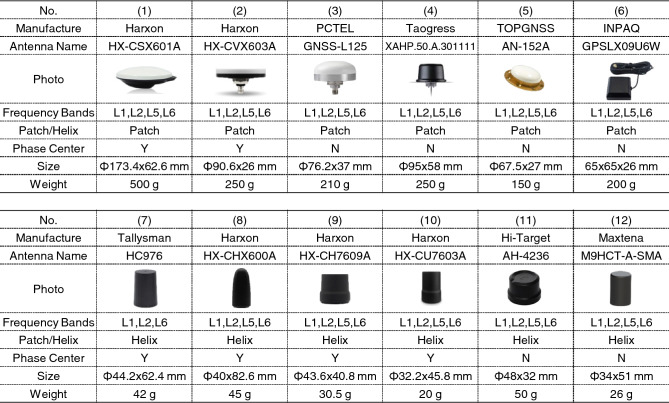
L6-compatible antennas to be evaluated. We selected 12 antennas. #1 shows a surveying antenna for comparison, and the others are compact and lightweight L6-compatible antennas weighing less than 250 g and with a diameter of less than 100 mm. The “Size” row in Figure 2 shows the diameter and height for cylindrical antennas and the three-sided size for rectangular antennas.

Figure 2 shows a list of the GNSS antennas evaluated in this study and their specifications. This study evaluated 12 antennas that support the L6 signal and are available for purchase worldwide in 2023. The Harxon CSX-601A in #1 is a survey GNSS antenna for comparison, and the other GNSS antennas were selected to be compact and lightweight (less than 250 g and less than 100 mm in diameter). Among the antennas evaluated, six were patch and six were helical.

As shown in Figure 2, the weight of the helical antennas was much lower. The patch antennas weighed over 200 g, whereas all the helical antennas were very light, with a weight of only a few tens of grams. In applications in which weight is an issue, helical antennas are more advantageous than patch antennas. In terms of antenna size, helical antennas have a smaller footprint but greater height than patch antennas. In addition, small and lightweight antennas are more likely to lack the phase-center offsets provided by the manufacturer. When such antennas are used in applications requiring high-precision positioning, the ARP and PCO must be determined by placing the antenna at a point with a known position and observing it. Among the small lightweight antennas, the Harxon and Tallysman antennas #2, #7, #8, #9, and #10 provide antenna PCOs to the ARPs.

## Static test

**Figure 3:**
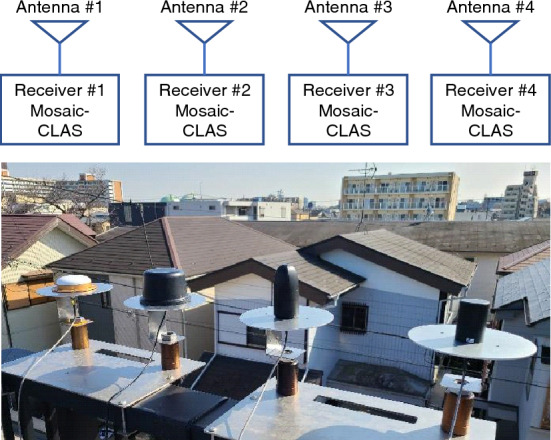
Experimental configuration and photographs of the antenna performance evaluation in a static test in an open-sky environment. GNSS observations are acquired simultaneously from four antennas for 24 hours. Each antenna is equipped with a 150-mm-diameter ground plane.

The L6 signal strength and L1 signal multipath characteristics were evaluated with respect to the elevation angle of the antenna using static tests in an open-sky environment. Figure 3 shows the configuration of the experiment and the photographs taken during the experiment. Four antennas were set up in an open-sky environment, and GNSS observations with a period of 1 Hz were acquired for 24 h using Septentrio mosaic-CLAS, which supported the reception of the QZSS CLAS signals. The four receivers were operated with exactly the same settings except for the antennas. After 24 h, the antennas were changed, and GNSS observations were acquired for the 12 antennas. The experiment was conducted between March 18 and 21, 2023, with an additional antenna added as an additional experiment on April 28 and 29, 2023. All data were acquired with a 15-cm-diameter metal ground plane to suppress multipath from ground reflections^25^, as shown in Figure 3, except for the CSX-601A survey antenna. In contrast to the survey antennas, antenna manufacturers recommend the use of antennas with an additional ground plane for small and lightweight antennas. Therefore, the same ground plane was used for all small antennas in this study to obtain data for fair comparison.

**Figure 4:**
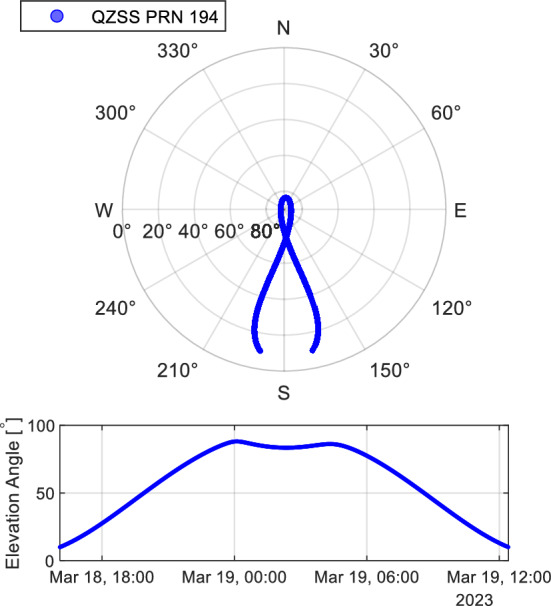
Satellite position and elevation angle of QZSS PRN194 during the static test. Data are collected for approximately 24 hours from the horizon from the time the satellite rises until it sets.

The L6D signal for CLAS broadcast from PRN 194 of the QZSS was used to evaluate the reception characteristics of the L6 signal. The satellite constellation and elevation angle of QZSS PRN 194 during the experiment are shown in Figure 4. The elevation angle started at 0″, and data were collected until it returned to 0° in approximately 24 h.

**Figure 5:**
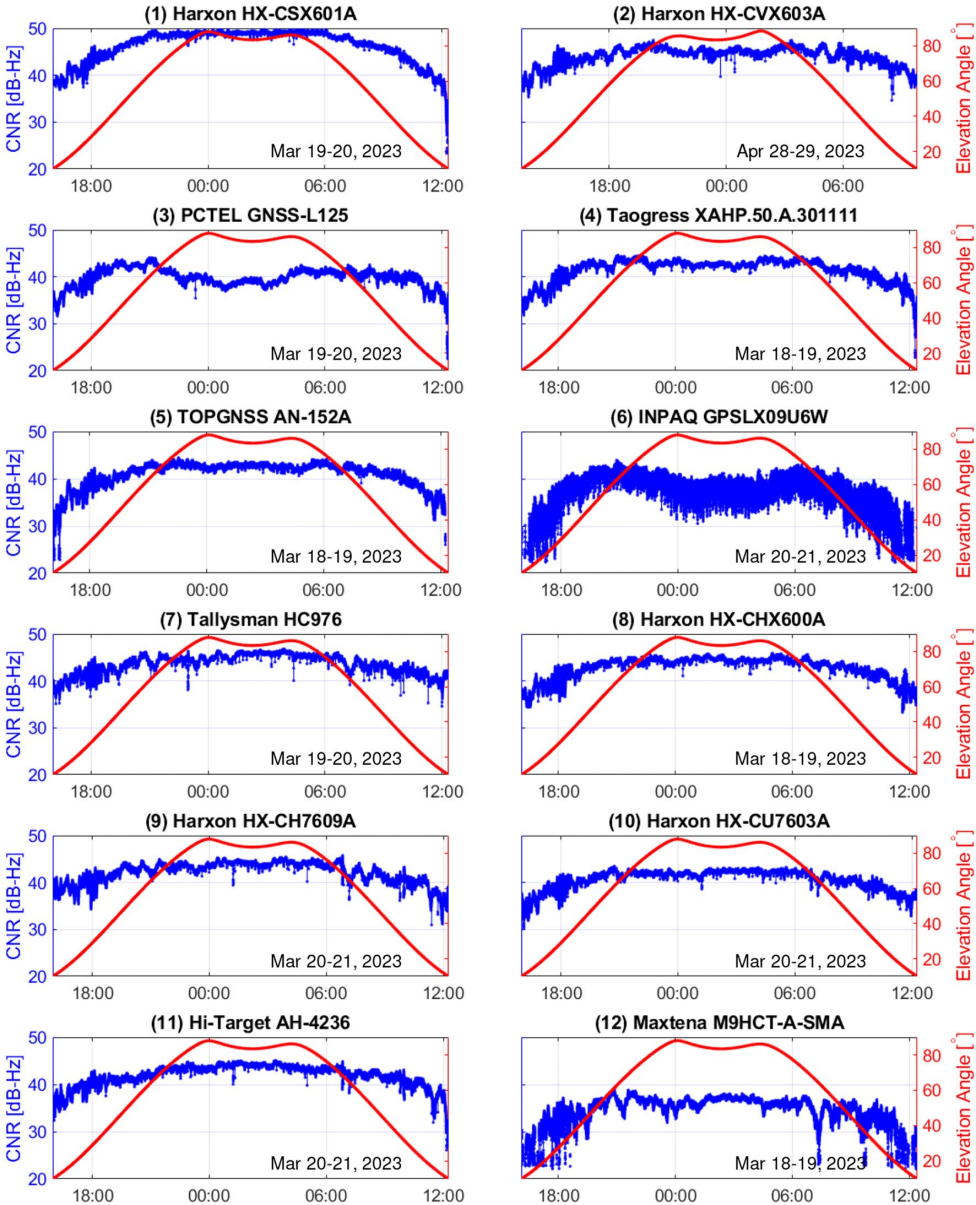
CNR of QZSS L6D signal and elevation angle of each antenna. A difference can be observed between the CNR at high and low elevation angles for each antenna. Some antennas cannot track the L6D signal stably at low elevation angles.

### Signal strength versus elevation angle

The carrier-to-noise ratio (CNR) of the L6D signal versus the satellite elevation angle for each antenna is shown in Fig. 5. The red line in Fig. 5 represents the elevation angle of the QZSS, and the blue line represents the CNR of the L6D signal at that time. As shown in Fig. 5, a large difference can be observed in the strength of the received L6 signal for each antenna. The survey antenna #1 (HX-CSX601A) has the highest L6 signal strength near the zenith; however, it has a lower signal strength at low elevation angles than the other antennas. For antennas #3 (GNSS-L125), #4 (XAHP.50.A.301111), #5 (AN-152A), #6 (GPSLX09U6W), #11 (AH-4236), and #12 (M9HCT-A-SMA), it was confirmed that L6 signal tracking was interrupted when CNR decreased at low elevation angles. In particular, the signal strength of antennas #6 and #12 was generally low, and the time during which the L6 signal could not be tracked was longer than that of the other antennas. In addition, when the signal strength was low at low elevation angles, cycle slips occurred frequently even when the L6 signal was tracked. Antenna 6 has a greater variation in signal strength than the other antennas. This fluctuation in the signal strength of antenna #6 was also observed in the repeat experiment, and the specific reason for this phenomenon is not known. In addition, a drop in CNR was observed several times. For example, this phenomenon occurred simultaneously at several antennas (antennas #9, #10, and #11) around 1:13 am on March 21. It is possible that interference of the GNSS signal by some external signal is the cause of this phenomenon. However, since this phenomenon occurred only a few times in a short period of time during the 24-hour data acquisition period, it does not have a significant impact on the statistical evaluation of the antennas.

**Figure 6:**
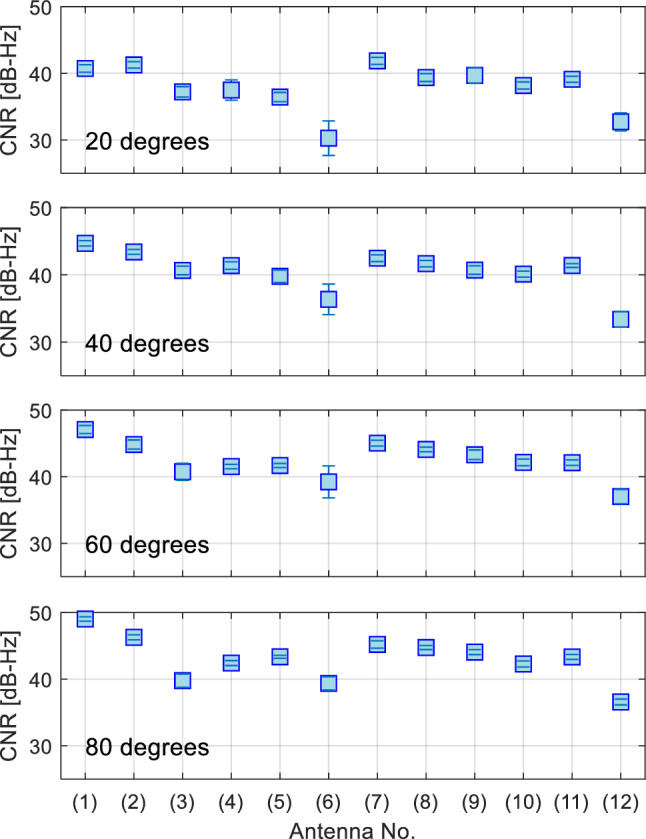
Comparison of the average CNR of the L6 signal and its standard deviation at satellite elevation angles of 20°, 40°, 60°, and 80° (top to bottom). A large difference is observed in the reception characteristics of the L6 signal at low elevation angles for each antenna.

Figure 6 shows a comparison of the average CNR and its standard deviation (1 sigma) for the satellite elevation angles of 80°, 60°, 40°, and 20°. The CNR at elevation angles in the range of +-1 degree of each reference elevation angle was extracted, and the mean and standard deviation were calculated. The survey antenna, antenna #1 (HX-CSX601A), stably receives the L6 signal at all elevation angles. Antenna #2 (HX-CVX603A) receives the L6 signal among the patch antennas, second only to the survey antenna. Helical antennas tend to have higher signal strength characteristics at low elevation angles than patch antennas. Among helical antennas, antennas #7 (HC976), #8 (HX-CHX600A), and #9 (HX-CH7609A) exhibit superior signal strength characteristics. Antennas #6 and #12 are not suitable for using the L6 signal for positioning in actual applications because the L6 signal cannot be stably tracked at an elevation angle of 20°. Antennas #2 and #7, which have high signal reception performance, are considered suitable for high-precision positioning applications using the L6 signal because the manufacturer also provides phase-center information relative to the ARP.

#### Multipath characteristics

The accuracy of the GNSS observations, including the multipath characteristics of the GNSS observations for each antenna, was evaluated. The multipath characteristics were evaluated using a multipath linear combination^1^. If the observed carrier phase at frequency band *A* is $\phi _A$ and observed pseudorange is $\rho_A$, and the observed carrier phase at frequency band *B* is $\rho_B$, the multipath combination $m_A$ is expressed by the following equation:

1 $$\begin{aligned} m_A=\rho _A-\phi _A-2k(\phi _A-\phi _B), \quad k=\frac{f_{B}^2}{f_{A}^2-f_{B}^2} \end{aligned}$$

where $f_A$ and $f_B$ denote the corresponding frequencies. $m_A$ includes code multipath error/noise, carrier multipath error/noise, frequency-dependent circuit delay, and carrier phase ambiguites. Carrier phase multipath error has a smaller absolute value than code multipath error, and carrier phase ambiguity and circuit delay shift $m_A$. Therefore, to evaluate the code multipath error, an offset is subtracted so that the average value of equation (1) is zero in the interval where no cycle slip flag is output by the receiver. As a result, the code multipath error and the carrier phase multipath errors remain, and the large code multipath error relative to the carrier phase multipath error can be evaluated for each target antenna. In QZSS CLAS and Galileo HAS, the L6 signal is used as a carrier for correction information, and the pseudorange and carrier phase of the L6 signal are not used for positioning. In high-precision positioning using correction information from satellites, it is the multipath characteristics of frequencies other than the L6 signal that affect the positioning accuracy. In this study, the multipath error of the L1 signal is evaluated.

**Figure 7:**
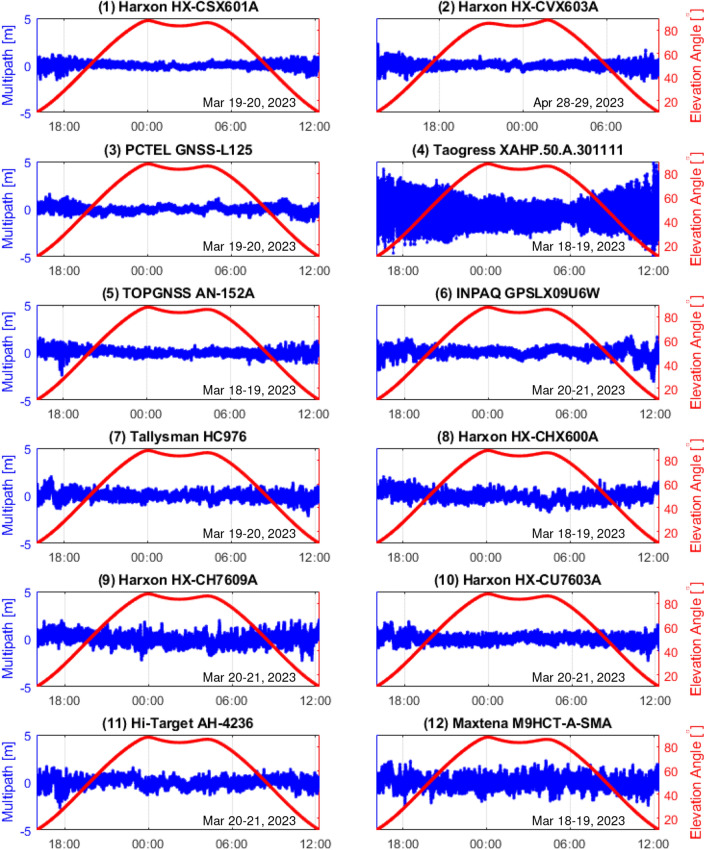
Pseudorange multipath values of the L1CA signal for each antenna. An offset is subtracted such that the average is zero. The multipath and its variation are small for the survey antenna, and the multipath error increases at low elevation angles.

As with the evaluation of signal strength, the QZSS signal at PRN 194 was used for the evaluation of multipath. Figure 7 shows the satellite elevation angle and magnitude of the linear multipath coupling for each antenna. As previously noted, an offset is subtracted such that the average is zero in the interval with no cycle slip. The elevation angle and magnitude of the multipath are related, and the multipath error varies significantly at low elevation angles. The survey antenna (antenna #1) has the most suppressed multipath at high elevation angles, whereas smaller and lighter antennas tend to have larger multipath errors. The multipath fluctuation of antenna #4 was greater than that of the other antennas throughout all elevation angles. Some antennas could not continuously track the L6 signal at low elevation angles, however, all antennas could continuously track the L1 and L2 signals.

**Figure 8:**
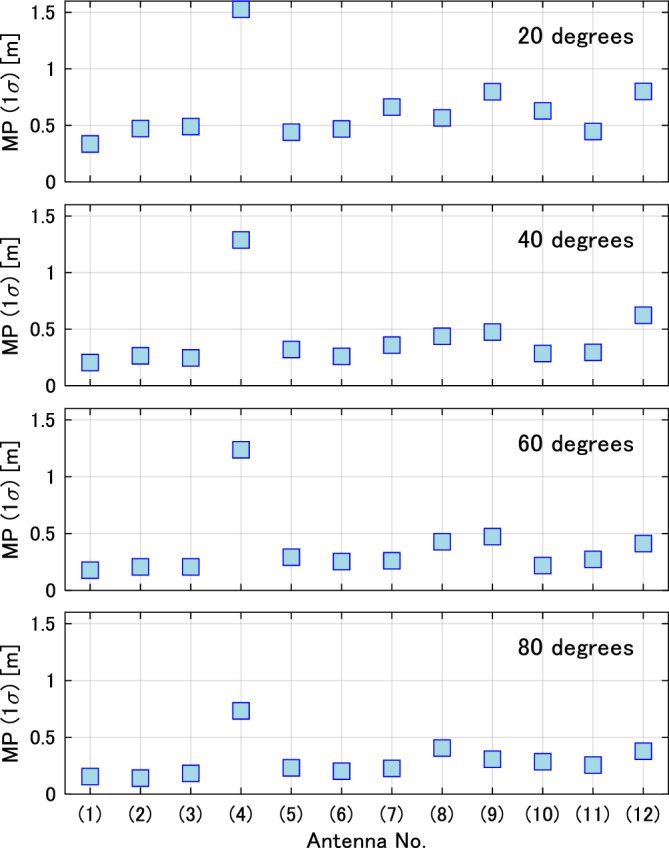
Standard deviation of pseudorange multipath of the L1CA signal (1 sigma) at satellite elevation angles of 20°, 40°, 60°, and 80°. The multipath error is large at low elevation angles, and the magnitude of the multipath error varies widely from antenna to antenna.

Figure 8 shows the standard deviation of the multipath error (1 sigma) for the satellite elevation angles of 80°, 60°, 40°, and 20°. A large variation can be observed in the multipath characteristics between the antennas. Antenna #1 for surveying has the lowest multipath error, especially at high elevation angles, confirming its high ranging performance. The helical antenna tends to have slightly larger multipath error than the patch antenna. Antenna #4 has a larger multipath variation than the other antennas. The multipath characteristics of antennas #2 and #7, which have good L6 signal reception characteristics, are excellent, although they are inferior to the antennas used for surveying.

## Kinematic test

**Figure 9:**
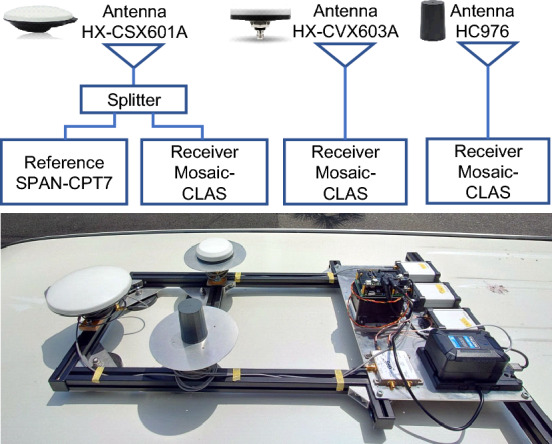
Experimental configuration and photographs of the positioning accuracy evaluation test using the QZSS L6D signal (CLAS) in the kinematic test. The NovAtel SPAN-CPT7 is used to acquire the reference position and evaluate the positioning accuracy of the three antennas that demonstrated high performance in the static test.

Three antennas, #1 (HX-CSX601A), #2 (HX-CVX603A), and #7 (HC976), which had high L6 signal strength in the static test, were used to compare the positioning accuracy of CLAS positioning using actual QZSS L6D signals when a vehicle was moving in a suburban area. Figure 9 shows the sensor configuration for the kinematic experiment and a photograph of the equipment, where three antennas were installed on the vehicle and PPP-RTK positioning by CLAS was performed simultaneously using Septentrio’s Mosaic-CLAS, as in the static test. For CLAS positioning, the PPP-RTK method is used to calculate the positioning solution by applying the correction information transmitted by the L6D signal to the L1 and L2 GNSS observations. The PPP-RTK engine used in the experiments was the one built into the mosaic-CLAS receiver, and the satellite systems used for CLAS positioning were GPS, Galileo, and QZSS only.

**Figure 10:**
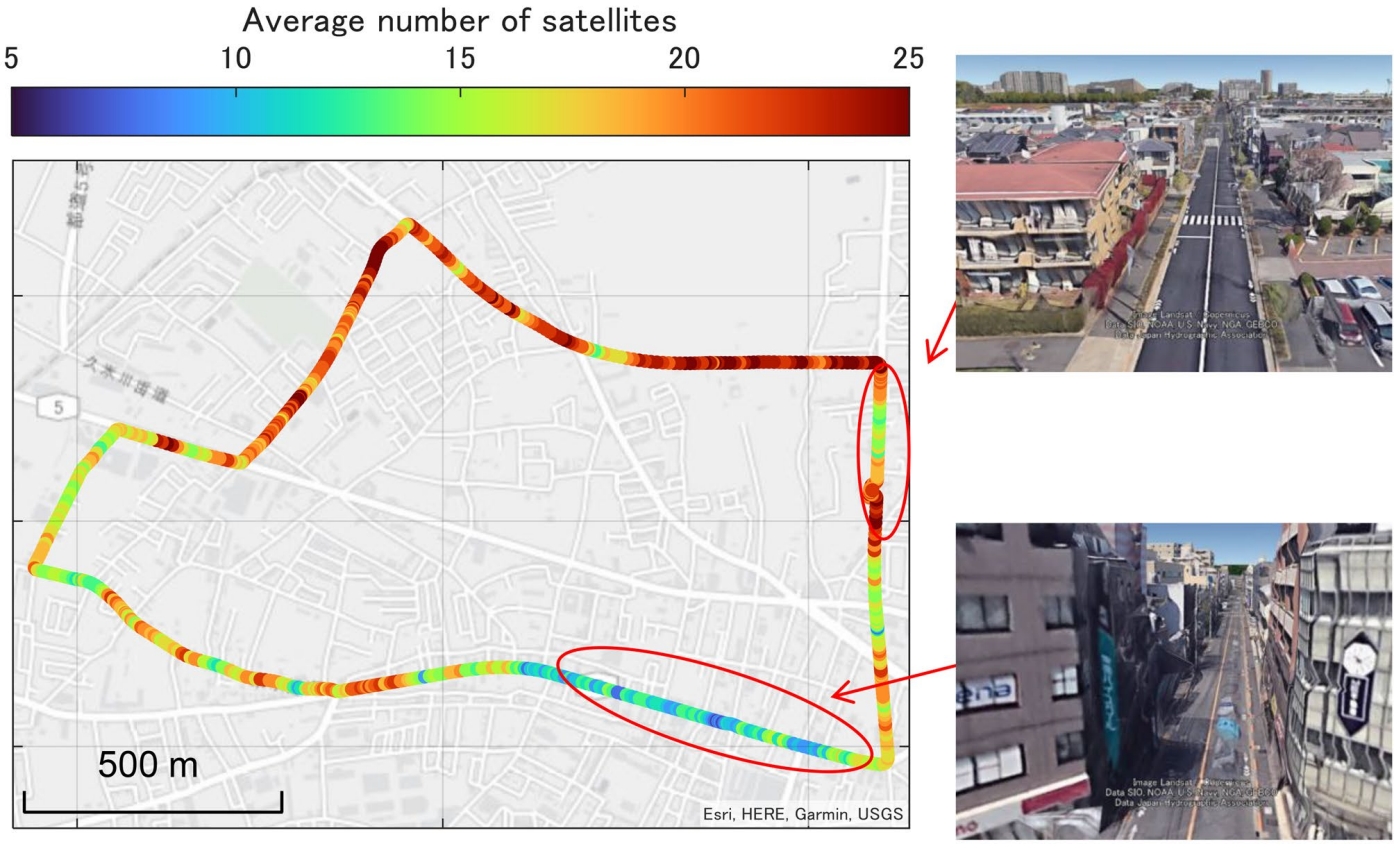
Vehicle trajectory and environment during the kinematic test. The color indicates the average number of received satellites. The environment is a typical suburban area where the satellite is sometimes obstructed by tall structures.

The position reference was NovAtel SPAN-CPT7, which is a combined GNSS/INS navigation system. Positioning accuracy is evaluated by driving the course shown in Fig. 10 several times at different times. As shown in Fig. 10, the driving environment was a suburban area. The colors of the plots in Fig. 10 indicate the average number of GNSS satellites received. Some of the driving sections are in an open environment lined with short buildings, and some sections are in an environment where the road is narrow and the surrounding buildings shield the GNSS signals. Experiments were conducted several times on the same route at different times to evaluate the positioning accuracy of CLAS between different antennas. During these experiments, the QZSS satellite transmitting the L6 signal was located near the zenith.

**Figure 11:**
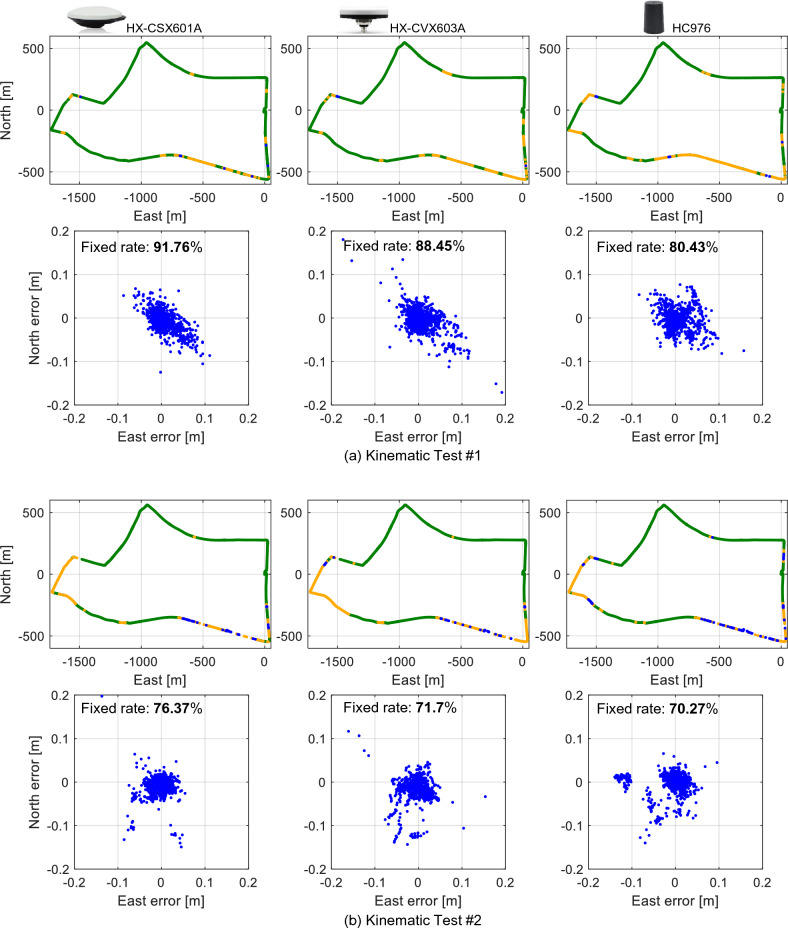
Comparison of positioning results from each antenna using CLAS in the kinematic test. The green dots in the figure indicate the PPP-RTK fixed solution, and the yellow dots indicate the float solution. The lower figure shows the horizontal error of each antenna compared to the reference.

Figures 11a,b show the positioning results for each antenna and the horizontal positioning error compared to the reference during two runs along the same route at different times. The green dots in the figure represent the fixed solution with the carrier-phase ambiguity resolved, and the yellow dots represent the float solution. The blue dots indicate code-based differential GNSS positioning solutions. Table 1 lists the L6 signal availability, carrier phase ambiguity fixed rate for each antenna, and the horizontal and vertical root mean square (RMS) errors of the fixed solutions. In both tests, the L6 signal availability was almost 100 % for all antennas. This is because QZSS was located almost at the zenith, and CLAS correction messages were received without interruption. In Test #1, the carrier phase ambiguity fixed rate for the surveying antenna exceeded 90 %, while the compact and lightweight antenna showed a slightly lower fixed rate. In Test #2, the overall fixed rate was lower than in Test #1, and as in Test #1, the surveying antenna had the highest fixed rate. The average number of satellites received over the entire travel time was 15.2 for Test #1 and 14.9 for Test #2. In Test #2, the number of visible satellites was lower in some sections, and the carrier phase ambiguity fixed rate was lower than in Test #1. In terms of positioning accuracy, the position could be estimated with centimeter-level accuracy at the points where a fixed solution was obtained in each antenna. The surveying antennas achieved slightly better positioning accuracy than the compact and lightweight antennas. However, the performance degradation was slight, and the carrier phase ambiguity fixed rate and positioning accuracy were sufficiently practical even with a compact and lightweight L6 signal-capable antenna. In conclusion, we confirmed that the QZSS CLAS can be used to estimate the position of a moving object, such as a vehicle, with centimeter-level accuracy using even compact and lightweight antennas with good reception characteristics for the L6 signal.

**Table 1:** Comparison of L6 availability, carrier phase ambiguity fixed rate, and positioning accuracy by antenna using CLAS in the kinematic test.

		HX-CSX601A	HX-CVX603A	HC976
Test #1	L6 availability %	99.92	99.92	99.92
	Fixed rate %	91.76	88.45	80.43
	Horizontal RMS error cm	3.45	4.06	3.65
	Vertical RMS error cm	5.97	7.70	6.42
Test #2	L6 availability %	100	99.92	99.92
	Fixed rate %	76.37	71.70	70.27
	Horizontal RMS error cm	3.09	4.07	4.62
	Vertical RMS error cm	4.27	6.05	7.35

## Conclusion

In this study, we evaluated the performance of small and lightweight L6-compatible antennas, which are expected to be used in future applications and are important for high-precision positioning using the L6 correction signal in a real environment. We compared small L6-compatible antennas from various perspectives, and evaluated the signal reception capability of L6 signals and multipath characteristics through static tests. In the kinematic test, we evaluated the accuracy of PPP-RTK using a small and lightweight antenna with CLAS broadcasted by the QZSS L6D signal. The results of this study are as follows:

- Even GNSS antennas that are catalog-compliant with the L6 signal have variations in the elevation angle-signal strength characteristics of the L6 signal, and some antennas cannot receive the L6 signal at low elevation angles. Therefore, each antenna should be evaluated individually to perform high-precision positioning using the L6 signals in actual applications.
- Small and lightweight GNSS antennas have poor signal strength and multipath characteristics compared to surveying antennas. The performance of patch and helical antennas is almost the same for small and lightweight antennas.
- PPP-RTK with QZSS CLAS can provide centimeter-level positioning, even when using small, lightweight L6-compatible antennas. This is useful for applications with space and weight limitations, such as drones.

The antenna evaluations in this study provide guidelines for antenna selection when high-precision positioning with L6 signals is required for various applications.

## Data Availability

The datasets generated during and/or analysed during the current study are available from the corresponding author on reasonable request.
